# The role of membrane excitability in pancreatic β-cell glucotoxicity

**DOI:** 10.1038/s41598-019-43452-8

**Published:** 2019-05-06

**Authors:** Zeenat A. Shyr, Zhiyu Wang, Nathaniel W. York, Colin G. Nichols, Maria S. Remedi

**Affiliations:** 1 Department of Medicine, Division of Endocrinology, Metabolism and Lipid Research, Washington University School of Medicine, 660 South Euclid Avenue, St. Louis, Missouri 63110 USA; 2 Department of Cell Biology and Physiology, Washington University School of Medicine, 660 South Euclid Avenue, St. Louis, Missouri 63110 USA; 3 Center for the Investigation of Membrane Excitability Diseases, Washington University School of Medicine, 660 South Euclid Avenue, St. Louis, Missouri 63110 USA; 4 Present Address: Endocrine Consultants Northwest, Franciscan Medical Group, 1628 South Mildred St. Suite 104, Tacoma, WA 98465 USA

**Keywords:** Cell signalling, Endocrine system and metabolic diseases

## Abstract

Persistent hyperglycemia is causally associated with pancreatic β-cell dysfunction and loss of pancreatic insulin. Glucose normally enhances β-cell excitability through inhibition of K_ATP_ channels, opening of voltage-dependent calcium channels, increased [Ca^2+^]_i_, which triggers insulin secretion. Glucose-dependent excitability is lost in islets from K_ATP_-knockout (K_ATP_-KO) mice, in which β-cells are permanently hyperexcited, [Ca^2+^]_i,_ is chronically elevated and insulin is constantly secreted. Mouse models of human neonatal diabetes in which K_ATP_ gain-of-function mutations are expressed in β-cells (K_ATP_-GOF) also lose the link between glucose metabolism and excitation-induced insulin secretion, but in this case K_ATP_-GOF β-cells are chronically underexcited, with permanently low [Ca^2+^]_i_ and lack of glucose-dependent insulin secretion. We used K_ATP_-GOF and K_ATP_-KO islets to examine the role of altered-excitability in glucotoxicity. Wild-type islets showed rapid loss of insulin content when chronically incubated in high-glucose, an effect that was reversed by subsequently switching to low glucose media. In contrast, hyperexcitable K_ATP_-KO islets lost insulin content in both low- and high-glucose, while underexcitable K_ATP_-GOF islets maintained insulin content in both conditions. Loss of insulin content in chronic excitability was replicated by pharmacological inhibition of K_ATP_ by glibenclamide, The effects of hyperexcitable and underexcitable islets on glucotoxicity observed in *in vivo* animal models are directly opposite to the effects observed *in vitro*: we clearly demonstrate here that *in vitro*, hyperexcitability is detrimental to islets whereas underexcitability is protective.

## Introduction

In the pancreatic β-cell, ATP-sensitive K^+^ (K_ATP_) channels play a critical role in coupling glucose metabolism to insulin secretion via control of membrane excitability, thereby maintaining blood glucose within a narrow physiologic range^1^. Increase in glucose metabolism leads to elevated intracellular ATP/ADP ratio and closure of K_ATP_ channels, with consequent membrane depolarization, leading to opening of voltage-dependent calcium channels and increased [Ca^2+^]_i_, which in turn triggers insulin secretion. Conversely, decrease in the metabolic signal opens K_ATP_ channels and suppresses the electrical trigger for insulin secretion^1^. Normally, the β-cell responds to chronic high glucose with a compensatory increase in β-cell mass to match the insulin secretory requirement. However, in genetically or otherwise predisposed individuals, β-cells are unable to sustain appropriate insulin secretory response and diabetes develops^2^. Persistent hyperglycemia may also lead to β-cell dysfunction and loss of insulin content^3^. Underlying mechanisms of this so-called glucotoxicity remain unclear, but the unique capacity of β-cells to increase oxidative phosphorylation in response to glucose availability makes them prone to increased reactive oxygen species production, which may underlie increased levels of oxidative stress markers and augmented apoptotic cell-death found in islets from type-2 diabetic organ donors^4–7^.

The importance of electrical activity in β-cell function is highlighted by the fact that K_ATP_ gain-of-function (GOF) mutations cause human Neonatal Diabetes Mellitus (NDM)^8^, K_ATP_ GOF polymorphisms are highly associated with type-2 diabetes^9^, and K_ATP_ loss-of-function (LOF) mutations underlie congenital hyperinsulinism (CH) (reviewed in^1,10,11^). Mouse models of NDM and CH due to genetically altered β-cell membrane excitability have been generated^12–19^. As expected, K_ATP_-GOF mice, with underexcitable β-cells and permanently low [Ca^2+^]_i_, show low circulating insulin levels and develop diabetes^12,20^. Importantly, as diabetes progresses, K_ATP_-GOF mice demonstrate a marked loss of insulin content, a typical secondary consequence of glucotoxicity^12^. However, the paradigmatic understanding that this glucotoxic loss of insulin content is the result of β-cell death is challenged by the demonstration of β-cell dedifferentiation, with no significant increase in apoptosis in pancreases from either diabetic mice or human type-2 diabetic organ donors^21–23^. Critically, we have demonstrated that this loss of β-cell mass in K_ATP_-GOF mice is caused by loss of mature β-cell identity and dedifferentiation to neurogenin3+/insulin negative cells, rather than apoptotic cell death^22^.

**Figure 1:**
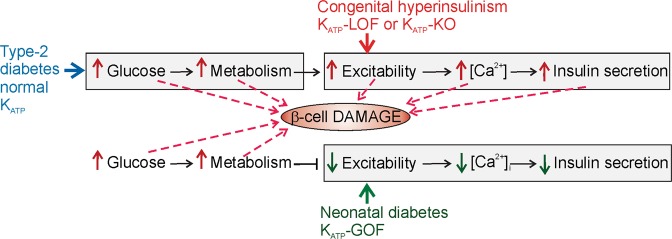
Schematic representation of the proposed key factors involved in pancreatic β-cell damage. In type-2 diabetes, hyperglycemia-induced hypermetabolism is constitutively coupled to membrane hyperexcitability, high [Ca^2+^]_i_ and insulin hypersecretion but in K_ATP_-LOF (congenital Hyperinsulinism) or K_ATP_-KO mice hypermetabolism is uncoupled from hyerexcitability and high [Ca^2+^]_i_, which are persistently high in these mice. Conversely, in K_ATP_-GOF induced neonatal diabetes, hyperglycemia-induced hypermetabolism is uncoupled from hyerexcitability and high [Ca^2+^]_i_ since they are constitutively low in these mice. Grey boxes represent the initial changes, and some of them have been attributed to cause beta-cell damage, as pointed by red arrows.

Chronic hyperglycemia will lead to hyperstimulated metabolism, which will be constitutively coupled to *hyper*excitability and chronically elevated [Ca^2+^]_i_, factors that have all been suggested to play a role in diabetic loss of β-cell function (Fig. 1). Moreover, decreased insulin secretion and mRNA and increased β-cell death have been demonstrated in multiple *in vitro* studies^2,3,24,25^. However, K_ATP_-LOF and K_ATP_-knockout (KO) mice, with chronically hyperexcitable β-cells and persistently elevated [Ca^2+^]_i_, do not show any obvious changes in insulin content or β-cell mass^15,16,18,26,27^, and K_ATP_-KO islets have been reported to be less susceptible to the toxic effects of high glucose, oxidative stress and death^28^. Conversely, as discussed, there is dramatic secondary loss of insulin content in K_ATP_-GOF mice that is not predicted as a direct consequence of their permanent *under*excitability and low [Ca^2+^]_i_.

In this study, we sought to determine the role of excitability in glucotoxic β-cell failure, and to ask whether this glucotoxicity is induced by hypermetabolism *per se*, or by the normally obligatorily coupled hyperexcitability and high [Ca^2+^]_i_. To do this, we have examined insulin content and secretion under glucotoxic conditions in islets isolated from mice in which the link between β-cell metabolism and excitability is lost. We have achieved this both genetically, using (i) islets from K_ATP_-GOF mice with *under*excitable β-cells and permanently low [Ca^2+^]_i_, and (ii) from K_ATP_-KO mice with *hyper*excitable β-cells with chronically elevated [Ca^2+^]_i_ (Fig. 1), and pharmacologically by treatment of wild type islets with chronic K_ATP_ channel activator (diazoxide) or inhibitor (glibenclamide). We demonstrate that underexcitability is protective against glucotoxic conditions *in vitro*, while hyperexcitability induces loss of insulin content even under low-glucose conditions. These results do not correlate with those obtained *in vivo*, where animal models of altered β-cell excitability show the opposite effects, highlighting the importance of whole body physiology.

## Results

### Morphological changes induced by high glucose in islets with normal or altered membrane excitability

**Figure 2:**
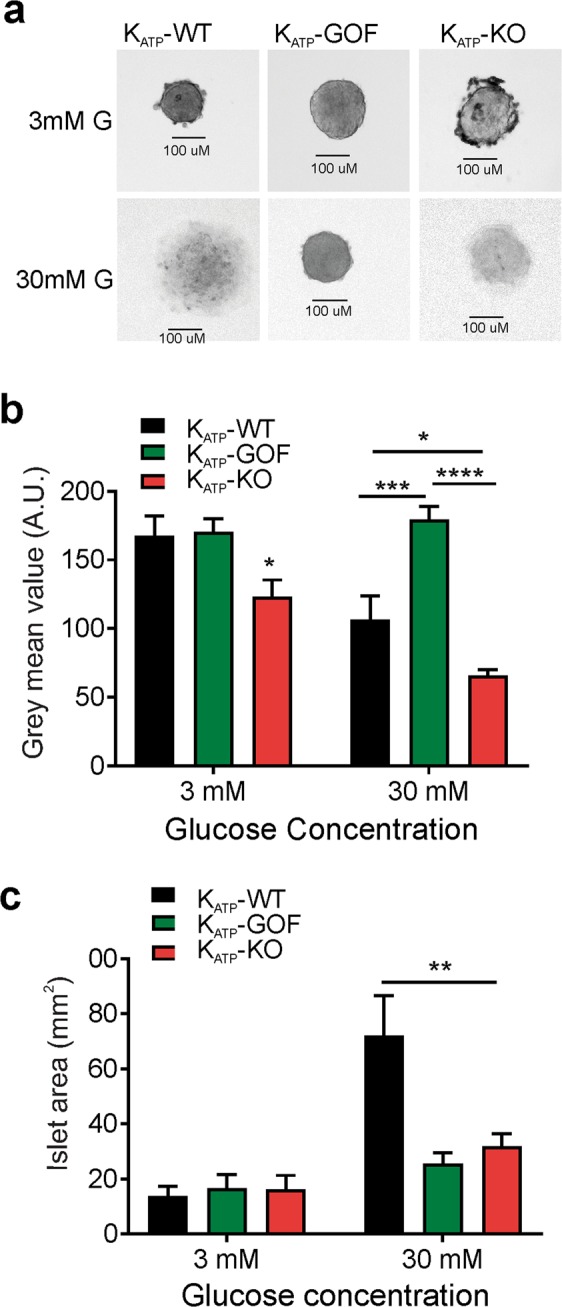
Islet Morphology. (**a**) Representative picture of pancreatic islets from K_ATP_-WT or genetically altered mice 9 days after incubation in low or high glucose. (**b**) Quantification of mean gray values (A.U) for islet translucency on K_ATP_-WT (black), K_ATP_-KO (red) or K_ATP_-GOF (green) islets chronically incubated in low- or high-glucose. n = 3 experiments from 9 independent mice, experiments were done in triplicates. Significant differences *p < 0.05, **p < 0.01, ***p < 0.001 and ****p < 0.0001 under the same condition; non-significant differences are not indicated in the figure. (**c**) Quantification of islet area (mm^2^) on K_ATP_-WT (black), K_ATP_-KO (red) or K_ATP_-GOF (green) islets chronically incubated in low- or high-glucose. n = 3 experiments from 9 independent mice, experiments were done in triplicates.

To directly test the influence of membrane excitability on glucotoxicity in β-cells, islets with normal (wild-type, K_ATP_-WT), or with genetically increased- (Kir6.2 knockout: K_ATP_-KO) or decreased-membrane excitability (Pdx-Cre/Kir6.2[K185Q, ΔN30] gain-of-function: K_ATP_-GOF) were isolated from adult mice and chronically incubated in low (3 mM) or high (30 mM, mimicking the glucose concentration that islets are exposed in K_ATP_-GOF mice) glucose for up to two weeks. Independent of the glucose concentration, or the genetically altered membrane excitability, the number of intact islets remaining in the dish did not change significantly over the period of time tested (at time 0: 30 ± 0 islets/dish on each genotype, after 10 day incubation: K_ATP_-WT: 27.4 ± 0.91 islets/dish, K_ATP_-GOF: 25.8 ± 1.2 islets/dish and K_ATP_-KO: 24.6 ± 1.6 islets/dish). However, K_ATP_-WT islets exposed to chronic high glucose became noticeably more translucent than islets incubated in low glucose (Fig. 2a,b) and were larger in diameter (Fig. 2c). K_ATP_-KO islets (with increased membrane excitability) incubated in low glucose were more translucent at low or high glucose compared to control and K_ATP_-GOF islets under the same conditions (Fig. 2a,b). Conversely, islets from K_ATP_-GOF mice (with decreased excitability) maintained their morphology and diameter (Fig. 2) suggesting that membrane underexcitability is protective.

### Electrophysiology of islets chronically incubated in low and high glucose

**Figure 3:**
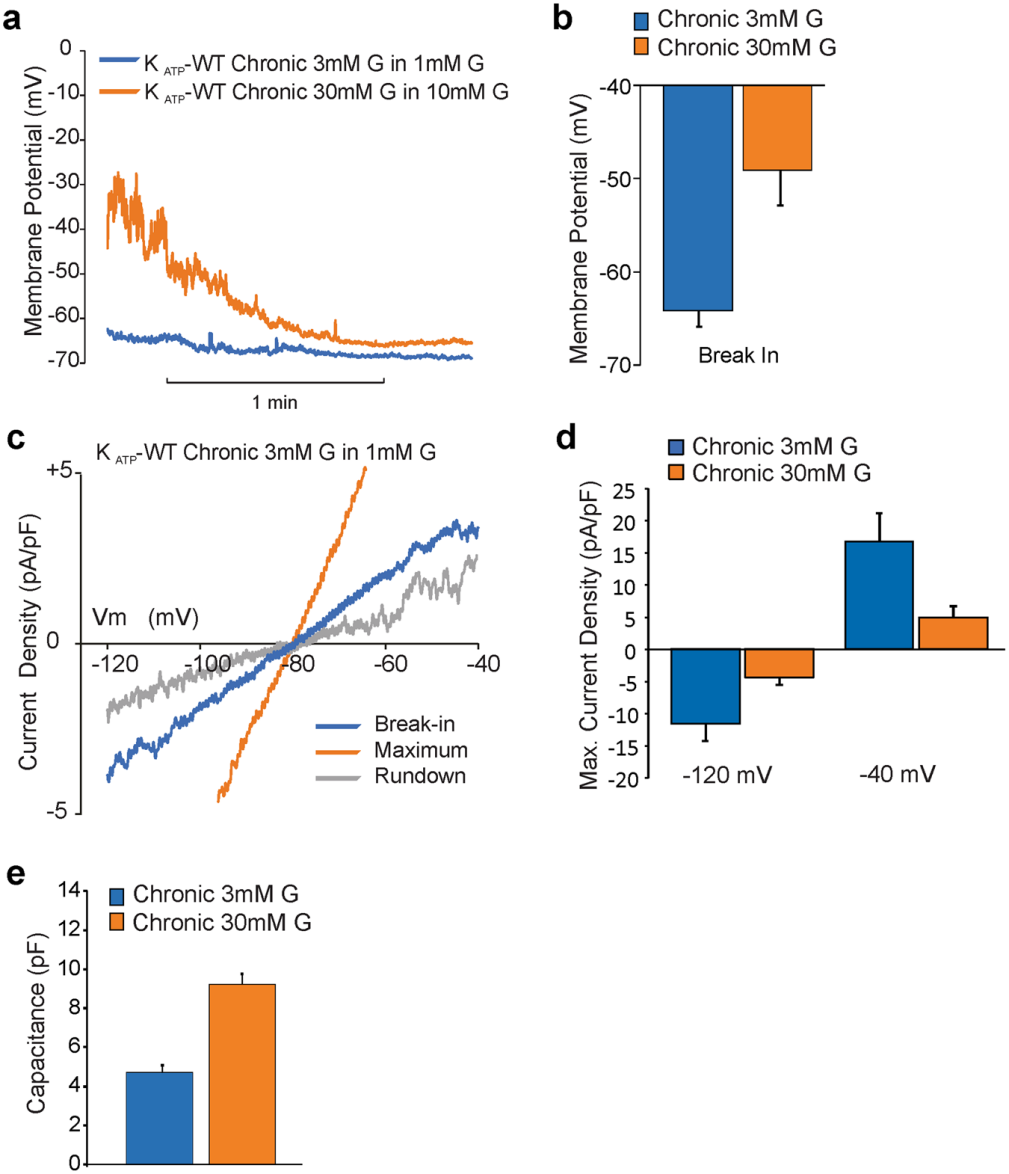
Beta-cell electrophysiology. (**a**) Representative traces of membrane potential during current clamp recordings from β-cells of K_ATP_-WT islets chronically incubated in 3 mM glucose (blue) or 30 mM glucose (orange) in bath solution containing 1 mM or 10 mM glucose, respectively. (**b**) Average membrane potential upon break into β-cells from islets chronically incubated in 3 mM or 30 mM glucose. (**c**) Representative traces of current density in β-cell from islets chronically incubated in 3 mM glucose showing break-in current (blue), current at maximal K_ATP_ activation (orange) and current after K_ATP_ rundown (grey). (**d**) Average current density at −120 and −40 mV during maximal K_ATP_ activation is shown from islets chronically incubated at 3 mM (n = 13, blue) and 30 mM (n = 23, orange) glucose. (**e**) Capacitance of β-cells from islets chronically incubated in 3 mM (blue) and 30 mM (orange) glucose.

We performed current clamp recordings of membrane potential and voltage clamp recording of membrane conductance and capacitance using β-cells isolated from K_ATP_-WT islets chronically incubated in low and high glucose. Figure 3 shows representative traces (a) and averages (b) of the membrane potential of β-cells (i) chronically incubated in low (3 mM) glucose following break-in in 1 mM glucose (blue), or (ii) chronically incubated in high (30 mM) glucose following break-in in 10 mM glucose (orange). As predicted for normal K_ATP_ behavior, the first are hyperpolarized (mean Vm = −64 ± 2 mV) due to active K_ATP_ channels (blue), whereas the second are depolarized (mean Vm = −49 ± 4 mV), but then hyperpolarize as K_ATP_ spontaneously activates following break-in (orange). Figure 3c shows voltage-clamp recording of membrane currents in response to voltage ramps (from −120 to −40 mV) from a cell chronically incubated in low (3 mM) glucose after break-in (in 1 mM glucose, blue), then after with spontaneous activation to maximum K_ATP_ (orange), then following subsequent K_ATP_ rundown (grey). The average maximum K_ATP_ current density in cells chronically incubated in 3 or 30 mM glucose is shown in Fig. 3d. K_ATP_ currents are present in both after the 10 day incubation period, although smaller in cells chronically incubated in high glucose. Figure 3e shows that cell capacitance, directly related to cell surface area, is approximately doubled in cells from islets chronically incubated in high glucose, correlating with the observed increased in islet area (Fig. 2c).

### Insulin content in islets from genetically altered mice chronically exposed to high glucose

**Figure 4:**
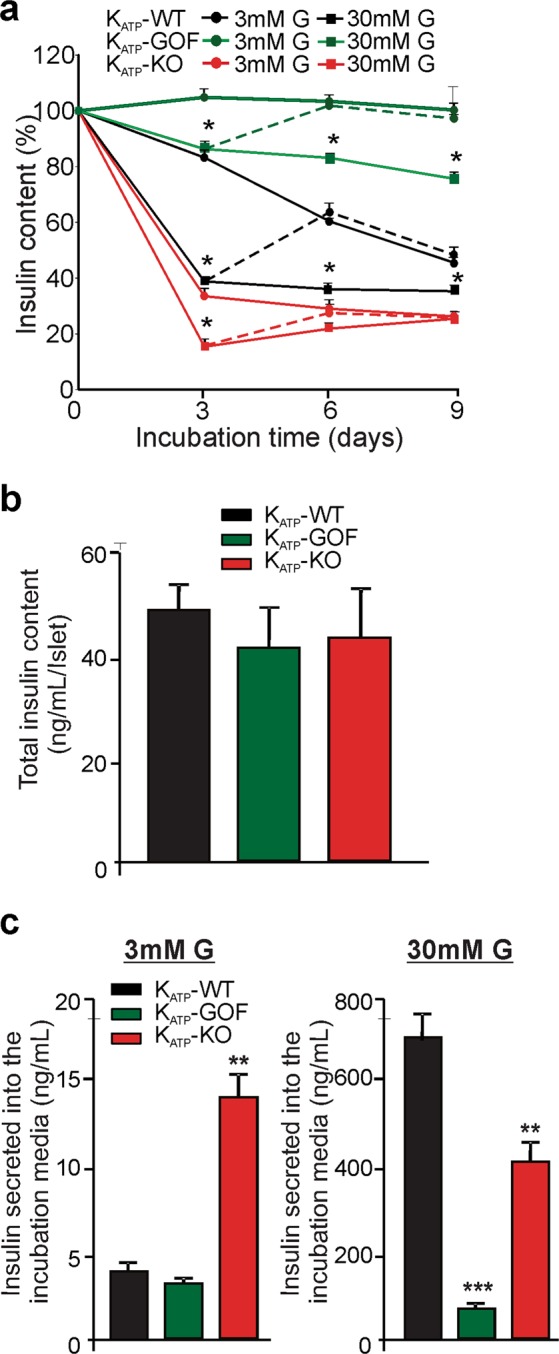
Glucotoxic changes in islet insulin content in vitro depend on membrane excitability. (**a**) Insulin content over time in islets from K_ATP_-WT (black), K_ATP_-KO (red) or K_ATP_-GOF (green) mice chronically incubated in 3 mM (circles, solid line) or 30 mM (squares, solid line) glucose, or when media has been switch from high to low glucose (dashed lines). n = 3 experiments from 6 independent mice, experiments were done in triplicates. Significant differences *p < 0.05 respect to control under the same condition; non-significant differences are not indicated in the figure. (**b**) Total insulin content per islet at day 0 of incubation from K_ATP_-WT (black), K_ATP_-KO (red) or K_ATP_-GOF (green) mice. (**c**) Insulin secreted into the media from K_ATP_-WT (black), K_ATP_-KO (red) or K_ATP_-GOF (green) islets chronically incubated in 3 mM or 30 mM glucose at day 3.

K_ATP_-WT islets incubated in high glucose demonstrated a marked and rapid reduction of initial insulin content (to ~40%), over the first 3 days, which was subsequently maintained with no further decline over longer incubation times (Fig. 4a, black circles and squares, solid lines). This early reduction in insulin content was dramatically reversed in islets that were switched back to low glucose after 3 days incubation in high glucose, indicating a reversible effect with no permanent damage (Fig. 4a, black dashed line). Conversely, K_ATP_-WT islets incubated in low glucose showed only a minimal reduction of insulin content over a period of 10 days. Strikingly, islets from K_ATP_-KO mice (with increased membrane excitability and chronically elevated [Ca^2+^]_i_^16^) also demonstrated a rapid (3 days) reduction of insulin content to a similar plateau as K_ATP_-WT islets, independent of whether incubated in low or high glucose (Fig. 4a, red circles and squares, solid lines). The reduction in insulin content observed in K_ATP_-KO islets at low glucose was slightly greater when incubated in high glucose, and again this was reversed to the level maintained in low glucose when the media was switched from high to low glucose (Fig. 4a, red dashed line).

As we previously described, K_ATP_-GOF mice (with reduced β-cell excitability^12^ and chronically low [Ca^2+^]_i_^20^), demonstrate severe diabetes within 2 weeks of tamoxifen induction of transgene expression. *In vivo*, this is followed by reduction in β-cell mass and insulin content due to dedifferentiation from mature β-cells to progenitor-like cells, secondary consequences of chronic systemic diabetes^12,22^, which include correlate loss of insulin and elevation of glucose. To specifically isolate the influence of electrical activity in glucotoxic conditions, islets from K_ATP_-GOF mice were harvested 10 days after tamoxifen-induction (i.e. prior to development of secondary consequences)^12^ and then chronically incubated in low or high glucose *in vitro*. In contrast to WT and K_ATP_-KO islets, insulin content of K_ATP_-GOF islets was completely preserved at low glucose, and only slightly reduced at high glucose (Fig. 4a, green circles and squares, solid lines), and again was completely reversed by switching the media from high to low glucose (Fig. 4a, green dashed line). These results indicate that membrane underexcitability protects from loss of insulin content in these glucotoxic conditions. K_ATP_-WT, K_ATP_-GOF and K_ATP_-KO islets all had similar total islet insulin content on day 0 (Fig. 4b). As predicted, islets from K_ATP_-KO mice demonstrate insulin hypersecretion when chronically incubated in low (3 mM) glucose, while K_ATP_-WT and K_ATP_-GOF do not secrete insulin in this condition (Fig. 4c, left). Moreover, while islets from K_ATP_-WT mice secrete insulin when incubated in 30 mM glucose media, K_ATP_-GOF show blunted secretion due to the mutation as expected (Fig. 4c, right).

### High glucose effect on islets with pharmacologically altered membrane excitability

**Figure 5:**
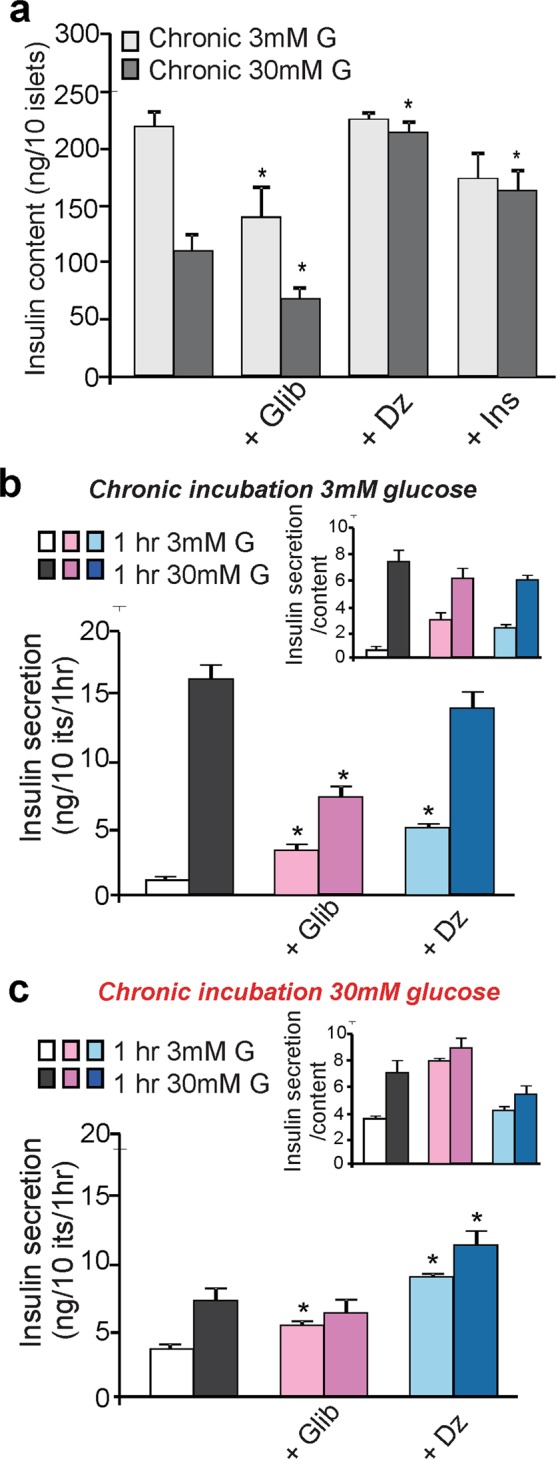
Chronic pharmacologic manipulation of membrane excitability alters insulin content and secretion. (**a**) Insulin content in WT islets incubated for 10 days in 3 mM and 30 mM glucose, or plus the addition of the K_ATP_ channel inhibitor glibanclamide (1 µM) or the activator diazoxide (250 mM), or insulin (20 nM). Significant differences *p < 0.05 with respect to control under the same condition, non-significant are not indicated in the figure. Insulin secretion response to acute low (light grey bars) or high (dark grey bars). Glucose stimulated insulin secretion on WT islets chronically exposed to low glucose (**b**) or high glucose (**c**) plus glibenclamide or diazoxide. Significant differences *p < 0.05 with respect to chronic glucose alone under the same stimulatory condition, non-significant differences are not indicated in the figures. Inserts represent insulin secretion as a fraction of content.

An alternative approach to manipulate membrane excitability is pharmacologically. WT islets were chronically incubated with the sulfonylurea glibenclamide (K_ATP_ channel inhibitor) to increase membrane excitability, or with diazoxide (K_ATP_ channel activator) to decrease membrane excitability, in parallel to the genetic approach above. As above, WT islets chronically incubated in high glucose demonstrated a marked decrease in insulin content compared to islets incubated in low glucose (Fig. 5a). Glibenclamide treatment caused a significant loss of insulin content in WT islets incubated in either low or high glucose (Fig. 5a). Conversely, diazoxide-treatment of WT islets resulted in preservation of insulin content in both low and high glucose conditions (Fig. 5a). These results mirror those obtained from K_ATP_-KO and K_ATP_-GOF islets, respectively (Fig. 4). To examine the consequences of insulin depletion *per se* in these experiments, exogenous insulin was added to WT islets incubated in low and high glucose. We demonstrate here that insulin prevented the high glucose-induced loss of insulin content (Fig. 5a).

### Effects of chronic pharmacologically increased or decreased excitability on glucose-dependent insulin secretion

We examined the insulin secretory response to glucose challenge in WT islets incubated for 10 days in low or high glucose, in the absence or presence of K_ATP_ channel inhibitors or activators. WT islets chronically incubated in low glucose secreted insulin normally in response to acute high glucose stimulation (Fig. 5b). However, WT islets that had been chronically incubated in high glucose showed an unexpectedly high basal insulin secretion in response to acute low glucose, but blunted response to acute high glucose (Fig. 5c). Importantly, WT islets chronically incubated in low or high glucose in the presence of glibenclamide also showed increased insulin secretion when acutely exposed to low glucose (Fig. 5b), and a marked decrease in insulin secretion when exposed to high glucose for one hour (Fig. 5c). Conversely, islets chronically incubated with diazoxide (K_ATP_ activator, which results in electrical ‘rest’) demonstrated both increased basal and glucose-stimulated insulin secretion, compared to islets exposed to glucose alone (Fig. 5b,c). When insulin secretion was calculated as a fraction of insulin content, it is clear that chronic glibenclamide acutely stimulates increased basal secretion, whereas diazoxide inhibits glucose-dependent secretion, in both cases (Fig. 5b,c, inserts).

### Proinsulin is increased in islets exposed to chronic high glucose

**Figure 6:**
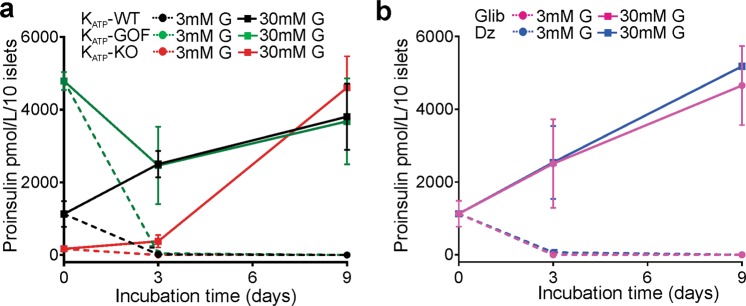
Changes in proinsulin content *in vitro* in islsts genetically altered or pharmacologically treated with K_ATP_ channel inhibitors and activators. (**a**) Proinsulin content at 0, 3 and 9 days on K_ATP_-WT (black), K_ATP_-KO (red) or K_ATP_-GOF (green) islets chronically incubated in low or high glucose. (**b**) Proinsulin content at 0, 3 and 9 days on WT islets chronically incubated in low or high glucose with or without the addition of glibencalmide (pink) or diazoxide (blue).

Because of the dramatic decrease in insulin content, we tested whether proinsulin biosynthesis was altered in genetically altered or pharmacologically treated islets. All islets exposed to chronic high glucose demonstrated a significant increase in proinsulin content, independent of the genotype (Fig. 6a) or pharmacologic treatment (Fig. 6b). At time 0, K_ATP_-KO islets showed lower proinsulin content than WT (Fig. 6a, red circles and squares), whereas K_ATP_-GOF islets demonstrated a markedly higher proinsulin level (Fig. 6a, green circles and squares). Conversely, all islets exposed to chronic low glucose demonstrated a significant decrease in proinsulin content over time, independent of the genotype (Fig. 6a) or pharmacologic treatment (Fig. 6b). These results demonstrate quite clearly that there is a positive effect of high glucose on proinsulin content, irrespective of membrane excitability.

## Discussion

### Chronic effects of hyperglycemia on islet function

Progressive deterioration in β-cell function, increased levels of oxidative stress markers, loss of insulin content and decreased β-cell mass *in vivo* have long been recognized in the pathogenesis of type-2 diabetes^4–6,24^, regardless of therapy^7,29^. Chronic hyperglycemia and elevated free fatty-acids negatively impact β-cell function and insulin secretion, but mechanisms underlying secondary loss of insulin remain unclear. In otherwise normal islets, chronic hyperglycemia will cause a persistent increase in membrane excitability and permanently elevated [Ca^2+^]_i_, potential key factors in deterioration of β-cell function ultimately leading to β-cell death^30,31^. However, this paradigm of β-cell death in diabetes has now been challenged by the demonstration of loss of pancreatic β-cell identity and cell dedifferentiation in both mouse and human diabetes^21–23,32^. The largely unknown and potentially detrimental effects of abnormally high or low [Ca^2+^]_i_ prompts us to ask the question: What role does membrane hyper- or hypo-excitability *per se*, and the consequently high or low [Ca^2+^]_i_, play in the deleterious effects of high glucose on islet function. By incubating islets from mice with genetically enhanced or reduced membrane excitability (i.e. K_ATP_-KO and K_ATP_-GOF mice respectively) in chronic high glucose *in vitro*, we can separate the role of excitability from otherwise linked *in vivo* factors in any glucotoxic response.

### The role of membrane excitability in glucotoxicity

The unique capacity of β-cells to increase oxidative phosphorylation in response to glucose availability makes them prone to increased reactive oxygen species production, which may lead to mitochondrial dysfunction and ultimately cell death. In WT islets, the marked reduction of insulin content and glucose-stimulated insulin secretion (GSIS) when chronically incubated in high glucose is consistent with earlier studies demonstrating that rodent β-cells adapt to chronic high glucose by reducing maximal GSIS, but without alterations in pancreatic islet mass^33^. The maintenance of insulin content in K_ATP_-GOF islets exposed to high glucose indicates that reduced membrane excitability protects them against such a loss of insulin content *in vitro*. In addition, this finding argues against any primary role of underexcitability and low [Ca^2+^]_i_^20^ in the loss of β-cell mass and especially insulin content that are observed in K_ATP_-GOF islets *in vivo*^12,22,34^.

Conversely, reduced insulin content in K_ATP_-KO mouse islets after chronic incubation in either low or high glucose suggests that chronic membrane hyperexcitability is a major determinant of insulin loss *in vitro*. Although there is no significant reduction in β-cell mass in normoglycemic K_ATP_-KO mice^15,16,35^, these results suggest that elevated [Ca^2+^]_i_ will be detrimental in the setting of hyperglycemia, consistent with studies indicating that high [Ca^2+^]_i_ is a key factor in reduction of β-cell functionality in diabetes^15,16,30,31,35^.

### Is increased membrane excitability, independent of altered metabolism, responsible for reduced insulin content?

Consistent with the idea that chronic β-cell hyperexcitability leads to a persistently elevated [Ca^2+^]_i,_ and insulin hypersecretion with depletion of insulin stores^24,36^, our *in vitro* data show that increasing membrane excitability, either genetically (K_ATP_-KO islets) or pharmacologically (glibenclamide-treated islets), does indeed lead to marked reduction in insulin content over ~3 days. Conversely, islets with genetically (K_ATP_-GOF islets) or pharmacologically (diazoxide-treated) reduced membrane excitability maintain insulin content over the full 10 days, even when they are chronically incubated in high glucose. Persistent inhibition of WT K_ATP_ channels with glibenclamide induces similar reduction of insulin content to that observed in K_ATP_-KO islets. These results correlate with studies demonstrating reduction of GSIS in rodent and human islets chronically incubated with sulfonylurea drugs, although these effects have only been observed *in vitro*, and not with consistency^36–38^. In addition, these results correlate with our previous observations that, in islets chronically exposed to glibenclamide, there is a mild increase in DNA synthesis; an effect that was completely abrogated by the presence of nifedipine (a calcium channel inhibitor), arguing that it is indeed driven by elevated [Ca^2+^]_i,_^39^. Moreover, our results are also consistent with those showing that islets chronically incubated in, or re-exposed to, sulfonylureas exhibited insulin secretion that occurs at lower [glucose] and accompanied by reduction in the maximal insulin response^40,41^.

Preserved insulin content and maintained GSIS (Fig. 4) in WT islets exposed to high glucose in the presence of diazoxide mimicked the effect observed in underexcited K_ATP_-GOF islets, supporting the hypothesis that ‘rest’ from hyperexcitability and therefore from chronically elevated [Ca^2+^]_i_ is protective against depletion of insulin content, and potentially against Ca^2+^-dependent apoptosis^24,36^. WT islets incubated in the presence of high glucose plus diazoxide have been reported to be more glucose sensitive, and to show a left-shift in glucose-stimulated insulin secretion, as well as a marked increase in DNA synthesis without changes in plasma K_ATP_ channels^39,42,43^. However, changes in β-cell oscillatory [Ca^2+^]_i_ and K_ATP_ conductance and a shift in glucose sensitivity have also been reported in islets incubated overnight with high glucose plus diazoxide^44^ which is consistent with altered K_ATP_ trafficking to the plasma membrane. Our results also correlate with decreased insulin content and secretion in human islets incubated in high glucose for 4 days, effects that were partially reversed by addition of diazoxide in the incubation media^45^.

The concept of deficient insulin stores as a contributing factor to β-cell dysfunction in type-2 diabetes arose many years ago, based on the observation that type-2 diabetic patients show restoration of insulin response to oral sulfonylurea stimulation after a period of treatment with diazoxide or insulin, in order to induce β-cell ‘rest’^46–49^. Diazoxide treatment has also been shown to improve subsequent β-cell secretory function in 90% pancreatectomized diabetic rats and in streptozotocin-diabetic rats^50^, and to reverse the marked loss of glucose-induced rise in [Ca^2+^]_i_ in human islets exposed to chronic high glucose^51^. Taken together, these *in vitro* and *in vivo* studies implicate electrical excitability-dependent loss of β-cell insulin content in high glucose conditions, which can be restored after exposure to low glucose and consequent suppression of excitation. Normally, islets do not accumulate large amounts of proinsulin since it is processed into mature insulin, which is then secreted. We began culturing islets from K_ATP_-GOF mice at day 10 post tamoxifen at which point these mice are already hyperglycemic (>30 mM blood glucose levels) due to lack of insulin secretion. This will drive enhanced glucose metabolism, and since proinsulin biosynthesis is induced in response to glucose metabolism, elevated proinsulin in these islets may be expected^52^. In the K_ATP_-KO model, since insulin is persistently secreted (reflected in accumulation of insulin in the media in low glucose condition), proinsulin accumulation is minimal. As expected, when any islets (controls, K_ATP_-GOF or K_ATP_-KO) are exposed to chronically high glucose, they showed increased proinsulin synthesis in response to elevated glucose in the media.

While precise mechanisms underlying the decrease in insulin content are not clear, our finding of increased proinsulin in all islets incubated in high glucose is consistent with inefficient conversion of proinsulin to insulin, potentially due to chronic glucose-induced oxidative and endoplasmic reticulum stress. It has been shown that, while glibenclamide increases and diazoxide decreases insulin secretion, there is no effect of these drugs on proinsulin biosynthesis (reviewed in^53^). These results are consistent with our observations that islets chronically incubated in high glucose, whether in the presence of either glibenclamide or diazoxide (which induce opposite effects on excitability and [Ca]_i_), leads to increased proinsulin, thus highlighting the effect of high glucose itself on proinsulin accumulation, rather than membrane excitability *per-se*. Our results also correlate with those demonstrating decreased proinsulin/insulin ratio in human islets treated with diazoxide at 11 mM glucose, which, by reducing excitability, preserves insulin content^45^. In obese mouse models of type-2 diabetes, islet proinsulin synthesis is typically increased and is accompanied by decreased mature insulin granules, due to dysfunctional insulin processing, effects that are reversed following incubation in low glucose media^54^. Consistent with the findings in rodent islets, human islets exposed to high glucose show accumulation and preferential secretion of proinsulin, secondary to depletion of mature insulin granules^55^.

### Exogenous insulin prevention of high glucose-induced loss of insulin content

We have previously demonstrated that inclusion of insulin in the incubation media can protect islets from loss of endogenous insulin content^39^. Preservation of insulin content in WT islets that were co-incubated with high glucose and insulin is consistent with studies that demonstrate better preservation of β-cell function in newly diagnosed type-2 diabetic patients subjected to intensive insulin therapy than in those treated with sulfonylureas^7,46^. It has also previously been shown that insulin can acutely induce membrane hyperpolarization by activation of K_ATP_ channels^56^, although such a mechanism seems unlikely to contribute to the chronic preservation of insulin in the current experiments. At higher concentrations, DNA synthesis and islet proliferation can be induced by exogenous insulin^39^, but this is not likely at the concentration used in the present study.

### *In vitro* versus *in vivo* models of glucotoxicity

We demonstrate here that chronic membrane hyperexcitability induced by high glucose conditions underlies loss of insulin content, and that membrane underexcitability protects islets from high glucose-induced loss of insulin content *in vitro*. It is important to note that pancreatic β-cells from mice with altered membrane excitability show the opposite long-term outcomes *in vivo*. We and others have previously demonstrated a marked reduction of insulin content, β-cell mass and insulin mRNA in islets from K_ATP_-GOF diabetic mice with reduced membrane excitability^12–14,22,34^. This is the result of chronic systemic diabetes and correlates with findings demonstrated in other forms of diabetes^21,33,57^. The primary mechanism for loss of insulin was shown to be pancreatic β-cell dedifferentiation to progenitor-like cells in the severely diabetic state, with re-differentiation to mature β-cells following lowering of blood glucose by intensive insulin therapy^22,33^. Conversely, islets with chronically increased membrane excitability and increased [Ca^2+^]_i_ (from K_ATP_-KO^15,16,18^ and K_ATP_-LOF mice^20,26^) show preserved insulin content *in vivo*. Moreover, chronic pharmacologic treatment with glibenclamide leading to increase in membrane excitability did not demonstrate any significant reduction in pancreatic islet insulin content *in vivo*^35^.

These differences observed *in vivo* versus *in vitro* may arise from the shorter duration of exposure to high glucose typically examined *in vitro*, including in this study, which might not be sufficient for the long-term consequences detected *in vivo* to occur. In addition, the *in vitro* absence of other nutrients such as amino acids and incretin hormones, which are known modulators of β-cell sensitivity to glucose, may contribute to *in vivo* consequences. We previously demonstrated that the *in vivo* environment is important for the islet response in K_ATP_-GOF mice. Severely diabetic K_ATP_-GOF mice show increased glucose metabolism at non-stimulatory glucose concentrations^20^, whereas K_ATP_-GOF mice treated acutely with glibenclamide at disease onset can enter a sustained remission and maintain near normoglycemia^34^, effects that are characteristic of other animal models of β-cell overstimulation and diabetes^34,35,58^. Finally, it is also important to note that the types of stresses that β-cells are exposed to *in vitro* (e.g. islet isolation from vascularization, loss of endothelial cells, and changes in intracellular matrix) are absent *in vivo* and could influence the response to high glucose exposure. Thus, other *in vivo* factors could also play a role in the marked loss of drug responsiveness, β-cell function deterioration, reduction of insulin content and loss of β-cell mass in diabetes^11^.

### β-cell rest from hypermetabolism, hyperexcitability or hypersecretion? What does ‘rest’ mean in potential recovery from β-cell dysfunction?

Finally, there has been much interest in the idea that exogenous insulin (and insulin plus diazoxide) treatment leads to β-cell ‘rest’, and that this permits restoration of β-cell function. It is unclear exactly what ‘rest’ means in this context: Does it mean rest from hyperstimulated metabolism, hyperexcitation, elevated [Ca^2+^]_i_, or from insulin secretion? Some studies clearly lean towards rest meaning rest from secretion. The present study provides one clear answer: our results show that rest from hyperexcitability (and its downstream consequences) is the key determinant of maintenance of insulin content *in vitro*. K_ATP_-GOF islets (or WT islets treated with diazoxide), which are permanently in the ‘resting’ state in terms of excitability, [Ca^2+^]_i_, and insulin secretion maintain insulin content even in the chronic metabolically stimulated state of hyperglycemia. Conversely, K_ATP_-KO islets (or WT islets treated with glibenclamide), which are chronically excited, lose insulin content rapidly in both high and low glucose conditions.

## Methods

### Mouse models of altered membrane electrical activity

All experiments were performed in compliance with the institutional guidelines of, and approved by, the Washington University Animal Studies Committee. K_ATP_ knockout^16^ and tamoxifen-inducible Pdx1^PB^Cre^ER^TM β-cell specific K_ATP_-GOF (Kir6.2 [K185Q, ΔN30])^12^ mutant mice were previously generated. Control littermates were used in all experiments.

### Pancreatic islet isolation

Mice were anesthetized with Isofluorane (0.2 ml) and killed by cervical dislocation, and the bile duct was cannulated and perfused with Hank’s solution (Sigma) containing collagenase (Collagenase Type XI, Sigma). The pancreas was removed and digested at 37 °C, hand shaken and washed in cold Hank’s solution. Islets were isolated by hand under a dissecting microscope and maintained overnight in CMRL-1066 (5.6 mM glucose) culture medium (GIBCO) supplemented with fetal calf serum (10%), penicillin (100 U/ml), and streptomycin (100μg/ml)^12^.

### Islet morphology measurements

Following overnight incubation in CMRL media containing 5.6 mM glucose, 30 islets per group were chronically incubated for 10 days in CMRL-1066 containing low (3 mM) or high (30 mM) glucose (experiment done in triplicates). On day 9, islets images were obtained using the Leica DMI 4000B inverted microscope (Lecia microsystems, IL) under bright field. Islet mean grey value and islet area were analyzed by Fiji (ImageJ).

### Electrophysiological experiments

Whole cell recordings were made using an Axopatch 200B amplifier and Digidata 1200 (Molecular Devices), as reported previously^59^. Voltage clamp recordings were performed using a ramp protocol from −120 mV to +40 mV with cells held at −70 mV during the inter-pulse interval. The bath solution was Tyrode’s solution contained 137 mM NaCl, 5.4 mM KCl, 1 mM MgCl_2_, 2 mM CaCl_2_, 0.33 mM NaH_2_PO_4_, 5 mM HEPES and 1 mM Glucose with pH adjusted to 7.4 mM with NaOH. The pipette solution contained 120 mM KCl, 10 mM HEPES and 1 mM K-EGTA, with pH adjusted to 7.4. For current clamp recordings current was held at 0pA. The bath solution used for current clamp recordings contained 10 mM glucose for cells from islets chronically incubated in 30 mM glucose and was unaltered for cells from islets chronically incubated at 3 mM glucose. Glass electrodes were pulled from Kimble-Chase 2502 plain capillary tubes using a P-87 puller (Sutter instruments). For voltage clamp recordings electrodes with 1–3 MΩ tips were used and for current clamp recordings 5–7 MΩ tips were used.

### Islet manipulation and measurement of insulin secretion and content

30 islets per group were incubated at low (3 mM) or high (30 mM) glucose and were collected at day 3, 6 and 9 for measurement of insulin content. Insulin secreted in the media under 3 and 30 mM glucose conditions was measured on day 3. For reversibility experiments, isolated islets were incubated with high glucose for several days and then the media was changed to low glucose for the time indicated in the figures. Additionally, wild-type islets were chronically incubated for 10 days in low or high glucose in the presence of 1 μM glibenclamide or 250 µM diazoxide (to block or activate K_ATP_, and thereby pharmacologically increase or decrease membrane excitability, respectively), or in the presence of 20 nM insulin. After the chronic treatment period, the islets were counted, and then pre-incubated in groups of 10 per well in 12 well plates in glucose-free CMRL-1066 plus 3 mM glucose, then incubated for 1 hour at 37 °C in CMRL-1066 plus low (3 mM) or high (30 mM) glucose for insulin secretion experiments. After the incubation period, the medium was removed and assayed for released insulin. Experiments were repeated in triplicate. For islet insulin content, groups of 10 islets were disrupted using ethanol-HCl extraction and sonicated on ice for estimation of insulin content. Insulin secretion and content were measured using either Rat Insulin radioimmunoassay (RIA, Millipore, St. Charles, MO)^22^ or Ultrasensitive Mouse Insulin Elisa kit (Crystal Chem, Elk Grove Village, IL)^60^ according to manufacturer’s procedure^22^.

### Measurement of proinsulin content in islets

Islets were incubated overnight in CMRL media containing 5.6 mM glucose prior to all experiments. On days 0, 3, and 9, replicates of 10 islets per genotype was collected in microcentrifuge tubes, washed with PBS and re-suspended in acid-ethanol extraction buffer. Proinsulin content was calculated by utilizing the Mouse Proinsulin Elisa (Mercodia, Salem NC).

### Statistics

Data are presented as mean ± SEM. Differences among groups were tested using analysis of variance (ANOVA) and post-hoc Duncan’s test. When only two groups were compared, unpaired t-tests were used to assess significance. *Indicates significant differences, with p < 0.05 respect to control condition. **Indicates significant differences p < 0.01, ***p < 0.001 and ****p < 0.0001.
